# Role of smooth muscle cells in vascular calcification: implications in atherosclerosis and arterial stiffness

**DOI:** 10.1093/cvr/cvy010

**Published:** 2018-03-05

**Authors:** Andrew L Durham, Mei Y Speer, Marta Scatena, Cecilia M Giachelli, Catherine M Shanahan

**Affiliations:** 1 Division of Cardiology, James Black Centre, Kings College London, Denmark Hill, London, SE5 9NU, UK;; 2 Department of Bioengineering, University of Washington, Seattle, WA 98195, USA

**Keywords:** Vascular calcification, Medial, Atheroschlerosis, Smooth muscle cells

## Abstract

Vascular calcification is associated with a significant increase in all-cause mortality and atherosclerotic plaque rupture. Calcification has been determined to be an active process driven in part by vascular smooth muscle cell (VSMC) transdifferentiation within the vascular wall. Historically, VSMC phenotype switching has been viewed as binary, with the cells able to adopt a physiological contractile phenotype or an alternate ‘synthetic’ phenotype in response to injury. More recent work, including lineage tracing has however revealed that VSMCs are able to adopt a number of phenotypes, including calcific (osteogenic, chondrocytic, and osteoclastic), adipogenic, and macrophagic phenotypes. Whilst the mechanisms that drive VSMC differentiation are still being elucidated it is becoming clear that medial calcification may differ in several ways from the intimal calcification seen in atherosclerotic lesions, including risk factors and specific drivers for VSMC phenotype changes and calcification. This article aims to compare and contrast the role of VSMCs in driving calcification in both atherosclerosis and in the vessel media focusing on the major drivers of calcification, including aging, uraemia, mechanical stress, oxidative stress, and inflammation. The review also discusses novel findings that have also brought attention to specific pro- and anti-calcifying proteins, extracellular vesicles, mitochondrial dysfunction, and a uraemic milieu as major determinants of vascular calcification.

## 1. Introduction

### 1.1 Vascular calcification

Vascular calcification, the deposition of hydroxyapatite mineral in the arterial wall, is linked to an increased risk of heart disease, stroke, atherosclerotic plaque rupture.^1^ Calcification occurs in both the intimal and medial layers of the arteries. Intimal calcification is linked to arterial obstruction and atherosclerotic plaque rupture. In contrast medial calcification is linked to vessel stiffness, systolic hypertension, and increased pulse wave velocity leading to increased diastolic dysfunction and heart failure.^2^ Whilst originally thought to be a passive process^3^ calcification of both the intimal and medial layers is an active and tightly regulated process, principally driven by the vascular smooth muscle cells (VSMCs).

Intimal and medial calcification are increased in patients with Type 1 (T1D) and Type 2 diabetes (T2D)/metabolic syndrome (MetS), chronic kidney disease (CKD), and postmenopausal women affected by osteoporosis.

Coronary arterial calcification (CAC) is an independent predictor for all-cause mortality independent of diabetic status.^4^ More than 70% of men and 50% of women with T1D are affected with coronary artery disease with a risk factor to develop CAC by their mid-forties.^5^ Cardiovascular and CAC risk in T1D are principally driven by hyperglycaemia; in contrast, the cause in T2D/MetS is multifactorial, featuring several factors largely absent in T1D such as obesity, hyperlipidaemia, and hypertension. There is, therefore, a need to study and develop different models to uncover specific mechanisms and therapeutic targets.

T2D/MetS, characterized by obesity, hyperglycaemia, hyperlipidaemia, and insulin resistance, remains a significant independent cardiovascular and arterial calcification risk factor even after adjusting for age, smoking, body mass index, and hypertension.^6^ Plaques within the coronary arteries of this patient group have larger necrotic cores and significantly greater inflammation than non-diabetic plaques.^6^ Additionally patients with T2D have more extensive lesion calcification in the coronary, carotid, and other arterial beds.^6^

In addition to accelerated CAC, patients with diabetes (T1D and T2D) develop extensive medial calcification of the peripheral arteries of the feet and legs. The mechanisms driving this localized calcification response are poorly understood and likely include novel factors in addition to hyperglycaemia, such as neuropathy.^7^

In the CKD population, active inducers of calcification include hypercalcaemia, inflammatory cytokines, oxidative stress, uraemic toxins, and elevated inorganic phosphate (Pi) or hyperphosphataemia.^8^^,^^9^ Importantly, clinical studies have shown that hyperphosphataemia is closely associated with advanced vascular calcification in CKD.^10^^,^^11^ Non-dialysis patients with CKD have an increased incidence of CAC compared with controls but less than that observed in patients with end stage renal disease and on dialysis. Several investigators found a graded relationship between severity of CKD and CAC score independent of conventional risk factors for atherosclerosis.^12^^,^^13^ Further, diabetic patients with coexistent CKD had a higher prevalence, greater extent, and more rapid progression of CAC.^14^

Numerous epidemiological studies have provided evidence of a link between osteoporosis and cardiovascular disease. The risk of coronary artery disease, stroke, and vascular calcification is higher in patients with a history of osteoporotic fracture or low bone mineral density than in non-osteoporotic patients.^15^^,^^16^ Vascular calcification involves cytokines and growth factors that also play a role in bone turnover, including pro-inflammatory cytokines [IL-6 and tumour necrosis factor-α (TNFα)], osteoprotegerin, sclerostin, matrix gamma-carboxyglutamic acid-rich (GLA) protein (MGP), and fibroblast growth factor (FGF)-23. Thus, aberrant levels of osteoprotegerin, sclerostin, or FGF-23 may explain and predict the occurrence of both osteoporotic fractures and cardiovascular events.^15^^,^^17^

### 1.2 Smooth muscle cells

A key cell type involved in vascular calcification is the smooth muscle (SM) cell. Smooth muscle cells are non-striated, non-voluntary, contractile cells,^18^ found in a variety of tissue types including the blood vessels, the trachea, the iris, the urinary bladder, and the digestive tract. Smooth muscle is essential for the optimal function of blood vessels, primarily maintaining blood pressure through contraction and relaxation in opposition to the heart.^19^ VSMCs also play a vital role in maintaining and remodelling the extracellular matrix (ECM) of blood vessels.^20^

**Figure 1:**
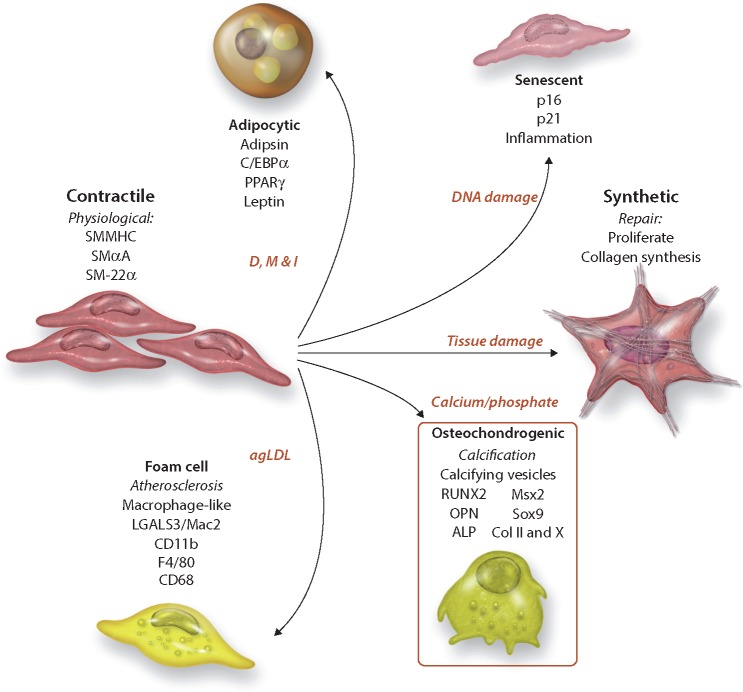
Diagram of possible VSMC phentoypes and their cellular/disease/organotypic origin. Diagrammatic representation of the spectrum of VSMC phenotypes identified during calcification (bold), their cellular markers and phenotypic drivers in red. Dexamethasone (D), Methylisobutylxanthine (M), and insulin (I).

In normal adult tissue, SM myocytes have a contractile phenotype: they proliferate slowly, are functionally contractile, respond to signals such as acetylcholine and norepinephrine and express a range of contractile proteins, including SM α-actin (SMαA), SM-22α, SM myosin heavy chains SM-1 and SM-2, calponin, and smoothelin. However, unlike other myocytes, SM cells are not terminally differentiated and display phenotypic plasticity.^21^ Smooth muscle cells can alter their phenotype in response to local cues, such as injury, and are capable of down-regulating contractile proteins, increasing proliferation, and remodelling the ECM to facilitate migration. Historically, this phenotypic transition from a contractile to what is termed a ‘synthetic’ state was viewed as a binary process with cells returning to the contractile state after repair had been completed. However, more recently it has become clear that SM cells are able to maintain a spectrum of phenotypes and can display features of osteoblasts, chondrocytes, adipocytes, and macrophage foam cells (*Figure 1*).

The change from the contractile to an osteo/chondrogenic phenotype is characterized by the development of calcifying vesicles, down-regulation of mineralization inhibitory molecules, and elaboration of a calcification prone matrix.^22^ This phenotype is accompanied by loss of SMC markers (SM22α and SM α-actin) and gain of osteochondrogenic markers [Runx2, SP7, osteopontin, osteocalcin, and alkaline phosphatase (ALP), Sox9, Type II, and X collagen (Col II and Col X)].

Much of the work to classify VSMC phenotypes has been carried out *in vitro*, stimulating the cells to drive differentiation along different lineages. VSMC cultured with aggregated low-density lipoprotein (agLDL) have down-regulated elastogenic capacity and increased macrophage foam cell markers, such as LGALS3/Mac2, CD11b, F4/80, and CD68.^23^^,^^24^ Similarly, VSMCs grown in apidogenic differentiation media develop adipocyte markers, such as adipsin, adipocyte fatty acid-binding protein, C/EBPalpha, peroxisome proliferator-activated receptor gamma (PPAR-γ), and leptin.^25^

**Figure 2:**
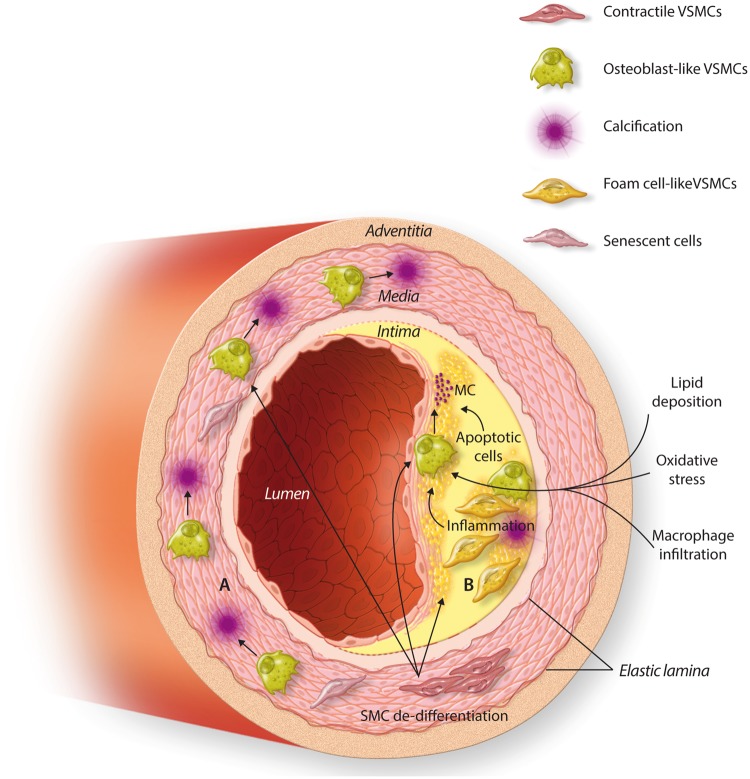
VSMC differentiation in intimal and medial calcification. (*A*) Within the medial layer the VSMC cells respond to osteogenic stimuli, e.g. prolonged uraemia, and differentiate into osteoblast-like cells. These subsequently produce macrocalcification deposits within the medial layer of the blood vessel causing stiffening of the vessel wall. (*B*) Atherosclerosis is characterized by lipid deposition between the intimal and medial layers of the blood vessel, which subsequently leads to macrophage infiltration, as well as the differentiation of VSMCs into foam cells. Inflammation, apoptosis, and oxidative stress subsequently lead to VSMC differentiation into osteoblast-like cells which, in turn, leads to microcalcification deposits within the intimal wall, weakening the structure of the wall and increasing the risk of plaque rupture.

Whilst VSMC plasticity is widely accepted the spectrum of phenotypes the cells are able to form and their relative importance in vascular calcification remains controversial. It is currently believed that VSMC phenotype switching during calcification varies depending on the location of calcification, for example inflammatory phenotypes developing during intimal rather than medial calcification (summarized in *Figure 2*). *In vivo* attempts to resolve this using lineage tracing experiments can be confounded by the changes in expression of cellular markers, such as the loss of SMαA and SM-22α,^26^^,^^27^ necessitating the use of advanced genetic fate mapping techniques. This challenge is further compounded by the ability of other cell types, such as multipotential vascular stem cells, adipose cells, fibroblasts, and macrophages to differentiate and gain VSMC marker expression.^28^ Recent studies showed that 40% of foam cells within advanced human coronary artery lesions express both the SMC marker ACTA2 and the macrophage marker CD68, although it is unclear if these represent VSMC-derived cells that have activated macrophage markers, are macrophages that have activated SMC markers, or neither.^29^

Importantly, many of these phenotypes are pathological and play roles in driving vascular disease processes. Therefore, understanding the environmental factors, transcriptional programmes, and signalling pathways that drive phenotypic change are key for future therapeutic strategies. The most intensively studied phenotypic transition of SMCs is osteo/chondrogenic conversion, which plays a major role in orchestrating vascular calcification of both the intima and media. This forms a paradigm for the role of SMC plasticity in disease and is the focus of this review.

### 1.3 Osteogenesis

Osteo/chondrogenesis is controlled by a number of defined transcriptional programmes regulated by physiological and mechanical cues. Osteogenic and chrondocytic differentiation from mesenchymal precursors is initially marked by the expression of both the transcription factors Sox9 and Runx2. The relative expression of Runx2 and Sox9 subsequently determines osteogenic or chondrogenic lineage, with Runx2 driving the osteogenic phenotype, whilst Sox9 binds to Runx2 and represses its actions.^30^ In the osteogenic phenotype, the Runx2 transcription factor in turn binds to downstream genes regulating bone development including ALP, Type 1 collagen, osteopontin, MMP9, and SP7.^31^

The transcription factor SP7, also known as osterix (OSX), and Wnt signalling further control development and drive an osteogenic rather than chrondrocytic phenotype. Loss of SP7 results in ectopic cartilage formation, highlighting its role in determining the osteo/chrondrocytic cell lineage. Wnt canonically activates β-catenin, which in turn enters the nucleus and binds to the DNA. β-Catenin is required for the progression from the Runx2^+^ stage to the Runx2^+^SP7^+^ stage and from Runx2^+^SP7^+^ cells to mature osteoblasts.

Other drivers of the osteogenic phenotype include activating transcription Factor 4 (ATF4), which is expressed in more mature osteocytes and the bone morphogenic proteins (BMPs). The BMPs are members of the transforming growth factor (TGF)-β family of proteins and BMP-2 has been shown to activate Runx2 in a number of cell types, as well a play a crucial role in bone repair.^32^

### 1.4 What drives calcification at different anatomical sites?

Although the VSMC phenotypic change associated with calcification is likely to be the same in both medial and intimal calcification what is clear is that there may be pronounced differences in the factors driving calcification at either site. For example, patients with renal failure develop accelerated medial calcification, which does not necessarily correlate with elevated atherosclerotic load. In other disease processes, such as diabetes, calcification of both the media and intima may be accelerated, however, the vascular beds affected may be completely independent. The characteristics associated with both intimal and medial calcification are summarized in *Table 1*. In the next sections, we will discuss the factors driving both intimal and medial calcification separately before comparing and contrasting these processes in an attempt to unravel core factors necessary for calcification.

**Table 1:** Summary of the characteristics of medial and intimal calcification

Characteristic	Medial	Intimal
Clinical complications	Arterial stiffness	Plaque rupture
	Increased pulse pressure	Myocardial infarction
	Increased pulse wave velocity	Stroke
	Surgical complications	
	Increased all-cause mortality	
Associated pathologies	Age	Atherosclerosis
	Diabetes	Hyperlipidaemia
	Renal failure	Metabolic syndrome
	Aortic aneurism	
VSMC phenotypes	Osteocytic	Osteocytic
	Chondrocytic	Chondrocytic
		Foam cell
Known drivers	Oxidative stress	Oxidative stress
	Apoptosis	Apoptosis
	Mitochondrial dysfunction	Mitochondrial dysfunction
	Mechanical stress	Mechanical stress
	Loss of inhibitors	Inflammation
	Uraemia	
	Senescence	

## 2. Arterial medial calcification

Arterial medial calcification (AMC) can occur in a range of conditions, including genetic disorders, aging, CKD, diabetes mellitus, dyslipidaemia, systemic lupus erythematous and hypervitaminosis D. Unlike atherosclerotic calcification, it may occur in the absence of lipid accumulation and inflammatory cell infiltration. Moreover, the diseases associated with medial calcification are diverse and often independent of atherosclerosis suggesting different processes drive VSMC change:

### 2.1 Loss of inhibitors

VSMCs can dynamically express a range of proteins that both inhibit and drive calcification, altering their transcriptional program as required, inducing Runx2, BMP2, and other osteogenic markers. These pro-osteogenic markers are balanced by inhibitors of calcification including MGP, osteopontin, BMP-7, and fetuin A.^33^ Decreased fetuin A and MGP levels are both found in CKD patients and low levels of inhibitors in patients are associated with increased risk of death.^34^

MGP is produced in both VSMCs and chondrocytes and serves as an inhibitor of BMP-2.^35^ It is proposed that the loss of MGP results in unopposed BMP-2 expression, enabling BMP-2 to drive changes in VSMC phenotype and subsequent calcification.^36^ Patients with Keutal syndrome, a rare autosomal recessive disorder caused by a loss of MGP function suffer from a variety of ailments, including abnormal cartilage and vascular calcification.^37^ Similarly murine models with *mgp*-knockout die after 6 weeks with massive arterial calcification.^35^ Decreased levels of MGP are found in diabetic patients^38^ and have been proposed to lead to unopposed BMP-2 driving AMC.

Fetuin A is derived from the liver and sequesters calcium and phosphate preventing calcification. Whilst not expressed by VSMCs, fetuin A is readily absorbed and concentrated in internal vesicles where it acts to inhibit the ability of vesicles to nucleate calcium phosphate precipitation.^39^ Fetuin A is decreased in patients suffering with vascular calcification and is an independent risk for patients undergoing dialysis.^40^

### 2.2 Cellular senescence

There is strong evidence linking medial calcification with aging^41^ and VSMC senescence, when cells no longer undergo mitosis but instead produce cytokines, growth factors, and proteases, termed the senescence associated secretory phenotype. VSMC senescence *in vitro* enhances calcification and osteogenic markers including ALP, collagen 1, and Runx2 expression.^42^ Furthermore in an aging model of mice senescence was linked to the emergence of medial calcification and Runx2 expressing osteoblast-like VSMCs.^42^

Hutchinson–Gilford progeria syndrome, a genetic disorder that leads to extreme aging, has also been linked to vascular calcification. Progeria is caused by a mutation in the *LMNA* gene, encoding the nuclear proteins Lamin-A/C,^43^ which results in the accumulation of a truncated form of pre-lamin A termed progerin. Pre-lamin A accumulates with age, VSMC senescence, and calcified arteries, including from children with CKD. Pre-lamin A accumulation disrupts the structural and functional integrity of the nuclear lamina and renders VSMCs more susceptible to mechanical stress.^44^

Due to the impact of aging and senescence, there has been much interest in the development of effective senolytic compounds, which will kill senescent cells in the body. Trials of senolytic compounds, such as Dasatinib and Quercetin, in mice led to reduced senescence cell markers in the medial layer of the vasculature, however, their effects on calcification have yet to be tested.^45^

### 2.3 Oxidative stress

Oxidative stress, such as reactive oxygen species (ROS), has been linked to vascular calcification. ROS accumulates in the vascular system with age^46^ and due to pathologies such as CKD.^47^ ROS accumulation is associated with increased *RUNX2* expression,^48^ which in turn may drive the osteocytic VSMC phenotype.

It has been suggested that diets high in antioxidant inducing compounds, such as resveratrol, can have a protective effect on vascular calcification.^49^ Initial research has indicated that antioxidants, such as N-acetylcysteine and tempol, can reduce VSMC calcification *in vitro* and in animal models^50^ but, as yet, no studies have been conducted to investigate the efficacy of antioxidants to prevent calcification in humans.

### 2.4 Mitochondrial dysfunction

The principle source of oxidative stress is free radical release from the mitochondria during oxidative phosphorylation, which can be increased by age, mitochondrial stress including aberrant calcium (Ca^2+^) influx. Myocytes require high glucolytic energy production for contraction, which is provided by the mitochondria and aerobic respiration. Changes in VSMC phenotype are linked to changes in mitochondrial metabolism, for example VSMC hyperproliferation, in pulmonary arterial hypertension is linked to raised mitochondrial metabolism.^51^

As well as energy production, the mitochondria plays an important role in mediating cell apoptosis, through the action of the caspase proteins. There is an increase of VSMC apoptosis, which was associated with osteogenic changes in the vessels of patients undergoing dialysis.^52^

Metformin, a common treatment for diabetes, is associated with reduced calcification and reduced osteogenic marker expression, such as ALP activity, Runx2 and BMP-2, in VSMCs *in vivo.*^53^ The mechanisms of metformin action are not fully understood but are linked to changes in glucose metabolism and insulin sensitivity, potentially acting through activating the adenosine monophosphate (AMP)-activated protein kinase (AMPK) pathway^54^ or the mitochondrial respiratory chain.^55^

The AMPK pathway acts as a central regulator of cellular energy homeostasis regulating cellular adenosine triphosphate (ATP) levels. AMPK is activated when bound by free adenosine diphosphate (ADP) and AMP, caused by cell energy depletion.^55^ AMPK puts cells into a low metabolic state, inhibiting protein synthesis. However, the role of the AMPK pathway in VSMC differentiation and calcification remains controversial and is linked to both driving and inhibiting calcification. Activation of the AMPK pathway can lead to VSMCs entering senescence^56^ and therefore drive calcification. Conversely activation of AMPK by resveratrol leads to change to the *in vitro* phenotype from synthetic to contractile.^57^ Similarly, AMPK activation has been shown to inhibit proliferation and migration.^58^

### 2.5 Mechanical stress

The haemodynamic conditions within the vasculature mean that VSMCs are under constant mechanical stress. These mechanical forces, such as transmural pressure, pulsatile pressure, and shear stress, have been linked to premature cellular aging, including through oxidative stress.^59^ The cell’s cytoskeleton plays a key role in mediating mechanotransduction within the cell.^60^ Disruption of mechanosignalling pathways in VSMCs, including the integrin–extracellular interface, actins, and cadherins, promotes dedifferentiation towards the synthetic phenotype. In turn, this dedifferentiation leads to a breakdown of the intimal and medial layers of the arteries, characterized as thoracic aortic aneurysm and dissection,^60^ which is itself associated with medial calcification.^61^ Yamanouchi *et al.*^62^ modified an existing mouse aneurism model with the addition of calcium phosphate, which led to vascular calcification associated with accelerated aneurism formation. This acceleration of calcification was even greater in apolipoprotein E deficient [ApoE(−/−)] mice; a common model of atherogenesis.^63^ There is evidence that BMP-2 induced osteogesis in mesenchymal stem cells (MSCs) is controlled by cell shape and cytoskeletal tension, via the Rho/Rho-associated protein kinase signalling pathway^64^ and this area requires further investigation in the context of medial calcification.

### 2.6 Uraemia

At physiological levels of calcium is a key signalling mediator in VSMC, regulating contraction, structure, and function.^65^ However, excess calcium, in levels corresponding to hypercalcaemic patients with CKD, can drive VSMC calcification including *in vivo*.^66^ VSMCs treated *in vitro* with uraemic serum undergo calcification; up-regulating osteopontin and losing their contractile markers.^67^

Extracellular calcium treated VSMCs show altered homoeostatic intracellular calcium and depletion of calcification inhibitors, such as MGP, leading to increased calcium loading and deposition within microvesicles.^68^ The exact mechanism through which high calcium drives phenotypic changes are unknown but have been linked to oxidative stress, DNA damage, and through direct signalling through the calcium uptake channels. The calcium receptor (CaR) is expressed in a range of tissue including VSMCs and in contractile VSMCs the CaR helps regulate vascular tone, proliferation and survival.^22^ In calcified tissue, CaR expression is down-regulated^69^ and ablation of CaR is correlated with increased calcification.^22^ At present, the mechanism through which the CaR influences VSMC calcification is unknown.

Elevated phosphate levels are also a common occurrence in CKD, and *in vitro* phosphate levels comparable to those in patients leads to dose-dependent calcification accompanied by a switch from contractile to osteochondrogenic phenotype.^70^ Calcium and phosphate added together act synergistically on VSMC *in vitro* to promote calcification, potentially though different mechanisms.^22^

Cells in the presence of phosphate also show in increase in ALP activity, mimicking differentiating osteoblasts, and hypertrophic chondrocytes. The increase in ALP activity in turn inactivates osteogenic inhibitors, such as dephosphorylating osteopontin and degrading pyrophosphate.^71^

Phosphate transport into cells is mediated by sodium-dependent phosphate (NaPi) co-transporters, of which the Type III NaPi co-transporters, PiT-1, and PiT-2, have been identified as the major transporters in VSMCs.^70^ Knockdown of PiT-1 in VSMCs inhibits phosphate-driven calcification and induction of osteogenic markers, such as Runx2.

In contrast, PiT-2 may have a protective role in vascular calcification. In idiopathic basal ganglia calcification, a rare neurodegenerative disease, loss of function mutations in PiT-2, have been identified as causal.^72^ Vascular calcification in these patients is most likely due to loss of protective effects of PiT-2 against arteriolar SMC calcification, as well as increased cerebrospinal fluid (CSF) phosphate levels resulting from PiT-2 deficiency in CSF generating brain epithelial.^73^

Vitamin D is also involved in calcium uptake and bone mineralization. Whilst normally beneficial excess vitamin D is linked to vascular calcification and in mouse models, the administration of high dose vitamin D leads to medial calcification, which is dependent on VSMC Runx2 expression.^74^ Similarly high doses of vitamin D in patients with CKD correlate with the severity of calcification.^75^ The principle mechanism of vitamin D-driven calcification appears to be the sequestering fetuin-A, although activation of FGF-23 and klotho enzyme, which are normally produced by osteoblasts, have also been implicated.^76^

Both high phosphate levels and vitamin D have been linked to premature cellular aging, especially in CKD. FGF-23- or Klotho-knockout mice have hypercalcaemia, hyperphosphataemia, and elevated levels of vitamin D and develop progeric symptoms have a shorter lifespan and exhibit vascular calcification and osteoporosis.^71^ Thus uraemia results in reduced inhibitors of calcification, increased oxidative stress, and cellular senescence in patients with CKD, making uraemia a potent driver of intimal calcification.

### 2.7 Cell death and damage

The damage and death of VSMCs plays a significant role in vascular calcification. *In vitro* uraemia models of calcification are associated with significant increases in VSMC apoptosis. It has been shown that inhibition of apoptosis, for example by caspase inhibitors, significantly reduces both calcifying vesicle release and calcification.^77^ Apoptosis occurs prior to calcification *in vitro* and apoptotic bodies are thought to contain high concentrations of calcium, which is ultimately deposited on the ECM causing calcification.

Following cell death, either by apoptosis or necrosis, not only is calcium released for the cell but also other cell contents including cellular DNA. Recently cell free DNA has been shown to precipitate calcium and phosphate and may initiate arterial calcification.^78^ This process may be of particular relevance to patients with end stage renal failure (ESRF), as not only does their condition lead to cell damage but also haemodialysis causes apoptosis both through direct contact with the membranes, or through activation of the complement cascade.^79^ Cell free DNA levels have been shown to be increased in patients suffering from ESRF, and this is further increased by dialysis, potentially increasing their risk of calcification.^79^

Cell damage may also result in the release of elastin fragments from the cell. Patients suffering from Marfan syndrome, a genetic disorder of the connective tissue, have extensive elastin fragmentation within their aortas. This damage to the arterial medial layer is a causal factor for microcalcification in Marfan patients, with the elastin fragments increasing ALP activity and reducing GLA expression.^80^

### 2.8 Future directions for AMC research

Whilst our knowledge of the role and drivers of VSMCs in medial calcification has significantly increased in recent years, nevertheless several key questions remain unanswered. Further research is required to better understand the drivers of the osteogenic phenotypic shift, and most importantly the timing of when this occurs. Currently, it is often assumed that phenotypic changes, such as *RUNX2* expression, precede calcification but this has yet to be conclusively demonstrated and may, in fact, be a cellular response to the osteogenic environment in which a VSMC finds itself. The other key question for current research is what is the nidus of calcification? Several suggestions, not mutually exclusive, for the nidus have been suggested apoptotic bodies, membrane-bound matrix vesicles (MVs), or hydroxyapatite nucleation on/within the collagen or elastin of the vessel wall.

## 3. Arterial intimal calcification

In contrast to AMC, patients with arterial intimal calcification (AIC) often have high serum pro-inflammatory cytokines, display hyperlipidaemia and/or metabolic syndrome, while calcium phosphate homeostasis is well-maintained. AIC is strongly correlated with atherosclerotic plaque burden,^81^ predicting adverse arterial events, such as plaque instability and risk of myocardial infarction,^82^^,^^83^ as well as risk of stroke.^84^ Superficial microcalcification within the fibrous caps of the atherosclerotic plaques are thought to promote local stress and chances of plaque rupture.^85^

Despite the recognized deleterious effects of AIC, there are currently *no* drug therapies available to prevent or treat this process, including statins, the mainstay of atherosclerosis treatment.^86^ Thus, recent studies have focused on identifying mechanisms and therapeutic targets that mediate AIC. These studies have identified cells of the VSMC lineage, osteochondrogenic differentiation, inflammation, oxidative stress, and apoptosis as potential mechanisms of atherosclerotic AIC as described later.

### 3.1 VSMCs in human atherosclerotic AIC

The historical view of VSMCs in atherosclerosis is that their migration to and proliferation within the intima contributes to initial atherosclerotic plaque formation, and at advanced stages, they form fibrous caps to stabilize vulnerable plaques.^87^ Accumulating evidence points to a substantial phenotypic plasticity of VSMC in response to injurious stimuli in the local microenvironment, linking the change of VSMCs to an osteochondrogenic phenotype with the development AIC. Detailed histological analyses of human coronary atherosclerotic AIC size and location have implicated VSMCs as major cellular orchestrators of AIC. Microcalcifications, typically <15 µm particles, were frequently observed in the fibrous cap, while macrocalcifications were found in the deep intima adjacent to internal elastic lamella and tunica media, all SMC-rich regions^85^^,^^88^ Similarly, molecules that initiate and regulate osteoblastic and chondrocytic differentiation (Runx2, BMP2, Msx2, osterix, and Sox9) are associated with atherosclerotic AIC.^89^^,^^90^ Cells with osteoblastic and/or chondrocytic properties commonly co-localize with calcium–phosphate deposits within atherosclerotic lesions.^88^^,^^90^^,^^91^

### 3.2 Genetic fate mapping of VSMCs in atherosclerotic AIC

The most direct evidence supporting osteochondrogenic differentiation of VSMC in AIC is from genetic lineage tracing studies in mouse models of atherosclerosis.^92^^,^^93^ These studies revealed that the majority of the osteochondrogenic precursor-like cells (∼75–88%) and almost all of the chondrocyte-like cells (∼98%) observed in atherosclerotic lesions were derived from VSMCs,^92^ implicating these cells as important mediators of AIC. The locations of the VSMC-derived cells in atherosclerotic lesions of these animals were almost identical to the locations of AIC occurring in human atherosclerotic lesions,^85^^,^^88^ mostly located in the fibrous cap of atheromas and areas of cartilaginous metaplasia and calcification. Like in human atherosclerotic AIC, these cells lost SMC marker protein expression, including SMMHC, SM22α, and smooth muscle actin (SMA), with the rare exception of some cells on the lumen side of the fibrous cap. Finally, at early time points, VSMC-derived cells were frequently observed to cluster in the deep intimal and inner medial layers, adjacent to elastic lamina breaks suggesting they were likely derived from vascular medial SMCs. These lineage studies are consistent with the electron microscopy study showing cells with hybrid SMC and chondrocyte properties, termed ‘myochondrocytes’, in human atherosclerotic lesions.^90^

### 3.3 Role of VSMC osteochondrogenic differentiation in atherosclerotic AIC

The critical role for VSMC phenotypic plasticity and osteochondrogenic differentiation in atherosclerotic AIC development is supported by a number of other studies. For example, deficiency of osteogenic regulators Msx1 and Msx2 in VSMCs of atherosclerotic *LDLr*^*−*^^*/*^^*−*^ mice inhibited osteogenic differentiation, resulting in reduced aortic calcification. Forced expression of osteogenic initiators, BMP2^94^ or S100A12,^95^ in VSMCs-activated osteoblastic differentiation and accelerated atherosclerotic AIC in *ApoE^−^^/^^−^* mice. Furthermore, removal of inhibitory *PPARγ*^96^ or *LRP6*^89^ from VSMCs augmented Wnt signalling, leading to increased VSMC osteoblastic and chondrocytic differentiation and AIC in atherosclerotic *LDLr^−^^/^^−^* mice. Finally, VSMC-specific depletion of Runx2 in atherosclerotic *LDLr^−^^/^^−^* mice resulted not only in dramatic inhibition of osteoblastic differentiation but also substantial reduction in chondrocyte maturation, leading to a 50% decrease of AIC in these animals.

Of interest, blocking VSMC osteochondrogenic differentiation affected lesion calcification but not systematic lipid metabolism, receptor activator of nuclear factor kappa-B ligand (RANKL) expression, monocyte/macrophage recruitment, or atherosclerotic lesion size.^74^ These findings identified for the first time a genetic separation between the calcific sclerotic process and the lipid-driven, atherogenic process, suggesting that different mechanisms regulate formation and progression of calcification and atheroma, respectively.^85^^,^^88^ Given these findings, it is tempting to speculate that the lack of effect of statins on AIC is due to major differences in aetiology between AIC and atherogenesis.

### 3.4 VSMC-derived macrophages and microcalcification of AIC

VSMCs differentiate not only into osteoblasts and chondrocytes during AIC but also into lipid-loaded, foam cell-like macrophages contributing directly to the advanced atherosclerotic plaque progression.^29^^,^^97^^,^^98^ In a confocal microscopy study of human coronary artery sections from hearts explanted at the time of transplantation, VSMCs were found to directly participate in the process of atheroma formation through macrophage foam cell formation, accounting for ∼18–40% CD68-positive cells observed in human advanced atherosclerotic lesions.^29^ These cells were spindle-shaped, positive for SM lineage marker protein SMA, and did not express myeloid cell markers, such as CD45. Further evidence was shown by a bioinformatics analysis that showed ∼35% of macrophage-like cells in human coronary atherosclerotic lesions were derived from VSMCs.^97^^,^^99^ Consistently, in a mouse model of atherosclerosis, cells of VSMC lineage were genetically labelled by an inducible VSMC-specific cre recombinase (*myh11-CreER^T^2*) and the eYFP Cre reporter transgenes. Approximately 30% of the total cells within the advanced atherosclerotic lesions were thus found to derive from VSMCs. Over 80% of the VSMC-derived cells lost the SM lineage marker proteins; some of them expressed macrophage marker protein Mac2 and MSC marker protein Sca1, contributing to ∼36% and of these populations.^97^ Interestingly, deletion of *KLF4*, a transcriptional repressor critical for VSMC differentiation resulted in a marked reduction in the number of VSMC-derived macrophage-like cells, atherosclerotic lesion size, and an increase in fibrous cap thickness of the atheroma.^97^ Contribution of VSMC-derived macrophages to osteochondrogenesis and AIC of atherosclerotic lesions was not determined in these studies, however, fibrous cap localization of macrophage-derived MVs and microcalcification^85^^,^^100^ raise this possibility and warrant of further exploration.

### 3.5 Inflammation and atherosclerotic AIC

Inflammation has long been recognized as a hallmark of atherosclerosis and was recently found to be associated with osteogenesis and AIC in human and animal cardiovasculature using spectrally distinct near-infrared fluorescent nanoparticle probes.^85^^,^^101^ Simultaneous administration of the fluorescent probes that target either macrophages or calcium–phosphate minerals to atherosclerotic mice, macrophages were found to coincide with osteogenic activity in the plaques, likely to produce extracellular MVs that serve as nucleating foci to initiate microcalcification.^100^ Although mechanisms and contributions of these extracellular vesicles to AIC remain to be clarified, superficial MVs in fibrous cap of atheroma create inhomogeneity of the plaque, which may contribute to the local plaque structural stress and vulnerability of the plaque.

Studies also determined a paracrine function of inflammatory cells, especially monocyte/macrophages, in regulation of VSMC phenotypic change and AIC.^102^^,^^103^ Monocyte/macrophages, lymphocytes, and dendritic cells infiltrate into atherosclerotic lesions during the early stages of atherogenesis. These cells produce pro-inflammatory cytokines and regulatory molecules that induce VSMC apoptosis or transdifferentiation into osteochondrogenic phenotypes, both of which contribute to mineral deposition in the plaques. For example, TNFα, released primarily by monocyte/macrophages, was found to be a key cytokines activating osteogenic program of VSMCs via Msx2-Wnt signalling.^104^ Likewise, receptor activator of NF-κB ligand (RankL) was found to increase the release of procalcific cytokines IL-6 by macrophages, promoting osteochondrogenic differentiation of VSMCs and AIC.^102^ Finally, macrophage secretion of IL-1β is correlated to VSMC osteochondrogenic differentiation and AIC progression.^103^

### 3.6 Oxidative stress

The relationship between oxidative stress and human atherosclerotic AIC has been well-documented.^105^^,^^106^ In symptomatic patients, coronary artery segments and stenotic aortic valves collected from explanted hearts, superoxide, and expression of the Nox family members were found to be enriched in atherosclerotic lesions, preferentially surrounding the calcifying foci, with Nox2 co-localized with the lesion macrophages and Nox4 co-localized with SMA-positive VSMCs,^107–109^ suggesting the involvement of both inflammatory macrophages and VSMCs.

The mechanisms of oxidative stress in atherosclerotic AIC are currently underexplored. It is well-known that during the early stages of atherosclerosis lipids and lipoproteins accumulate in the sub-endothelial space of arterial wall, undergoing oxidation by ROS produced during the metabolic activities of surrounding cells.^87^ The resulting oxidative products may activate the NF-κB-RankL pathways provoking the production of procalcific cytokines, such as TNFα, IL-6, and IL-1β,^102–104^ or induce mitochondrial DNA damage^87^ and apoptosis of surrounding cells, such as VSMCs,^87^ both of which contribute to the development of AIC. Indeed, exposure of VSMCs to oxLDL or hydrogen peroxide were found to induce osteoblastic differentiation of these cells^48^^,^^110^^,^^111^, likely mediated through the osteochondrogenic transcription factor Runx2 via AKT signalling.^48^ Finally, in a mouse model of atherosclerosis, global knockout of Nox2 nicotinamide adenine dinucleotide phosphate (NADPH) oxidase markedly reduced aortic superoxide production and plaque size,^112^ highlighting of the importance of ROS in atherogenesis.

### 3.7 SM apoptosis

VSMC apoptosis can be detected in human atherosclerotic lesions, possibly induced by lesion macrophages via death ligand and receptor interaction and oxidative stress.^87^ Apoptosis promotes matrix calcification, primarily through the release of the calcifying membrane-bound MVs, such as apoptotic bodies,^113^ acting as nucleation sites of calcification in blood vessels.^68^^,^^113^ Vesicles isolated from normal arteries are less efficient in accumulating calcium compared with those isolated from calcified human atherosclerotic vessels.^114^ Differences between the apoptotic bodies and MVs are underexplored, however, the biogenesis of these vesicles was found to be important in calcification, and the calcium-dependent loss of inhibitors, such as MGP,^115^ and the expression and redistribution of phosphatidylserine and annexin A6 complexes were critical for the vesicles to nucleate hydroxyapatite on the vesicle membrane.^68^

How calcifying MV production is regulated remains to be explored. One pathway receiving attention is autophagy, an adaptive stress response of cells to remove unnecessary or dysfunctional cellular components thereby maintaining intracellular homeostasis. Dai *et al.*^116^ recently determined autophagy as protective mechanisms against vascular calcification, possibly through the inhibition of apoptosis and MV release. Oxidative stress, provoked by high phosphate in this study, was determined as a critical mediator driving the adaptive autophagic response of VSMC stress by procalcific, high phosphate conditions. Inhibition of autophagy, either through a targeted inhibitor or siRNA approach, enhanced phosphate-mediated MV release, thereby exacerbating VSMC calcification.^116^

### 3.8 Future directions for AIC research

Risk factors for atherosclerotic AIC include aging, diabetes, metabolic syndrome, hyperlipidaemia, inflammation, and oxidative stress. Strong evidence supports a critical role for VSMC in the aetiology of AIC. Common mechanisms by which VSMC may contribute to AIC include phenotypic conversion/transdifferentiation to osteochondrogenic and macrophagic lineages capable of releasing calcifiable extracellular vesicles and apoptotic bodies, producing calcification prone-collagen and elastin matrices, and regulating the production of procalcific molecules and calcification inhibitors. Further studies to determine how specific risk factors modulate these mechanisms to promote AIC is greatly needed and could pave the way to future therapies.

## 4. Conclusions

It has become increasing clear in recent years that arterial calcification is not a passive process but involves active reprogramming of VSMCs by local environmental cues into a dynamic range of phenotypes. These local cues are different within atherosclerotic calcification, where the primary drivers are inflammation, oxidative stress, and apoptosis and medial calcification, which is associated with aging, senescence, uraemia, and high serum calcium and phosphate levels.

Whilst the drivers of phenotypic change are largely distinct, the phenotypic changes share common features, such as increased Runx2 expression and extracellular vesicle calcification, leading to speculation as to whether these distinct environmental cues act on a single or distinct intracellular signalling cascades to drive cellular reprogramming and phenotype switching.

Nevertheless increased understanding of VSMC phenotype switching offers the best chance of identifying novel therapeutic targets to help prevent the currently untreatable arterial calcification.

**Conflict of interest:** none declared.

## Funding

ALD and CMS were supported by the British Heart Foundation (BHF) BHF Programme Grant RG/17/2/32808 awarded to CMS. MYS and CMG were supported by National Institutes of Health grants R35HL139602, R01HL081785, R01HL62329 and R01HL114611. MS was supported by NIH grants R35HL139602, R01HL114611 and R01DK094434.
